# Clinical impact of newly developed atrial fibrillation complicated with longstanding ventricular fibrillation during left ventricular assist device support: A case report

**DOI:** 10.1186/s12872-019-1132-1

**Published:** 2019-06-21

**Authors:** Chie Bujo, Eisuke Amiya, Masaru Hatano, Masaki Tsuji, Hisataka Maki, Yumiko Hosoya, Emi Fujii, Tatsuya Kamon, Toshiya Kojima, Kan Nawata, Osamu Kinoshita, Mitsutoshi Kimura, Minoru Ono, Issei Komuro

**Affiliations:** 1 Department of Cardiovascular Medicine, Graduate School of Medicine, The University of Tokyo, Hongo 7-3-1, Bunkyo-ku, Tokyo, 113-8655 Japan; 2 Department of Cardiac Surgery, Graduate School of Medicine, The University of Tokyo, Tokyo, Japan; 3 Department of Therapeutic Strategy for Heart Failure, Graduate School of Medicine, The University of Tokyo, Tokyo, Japan

**Keywords:** Left ventricular assist device, Right heart failure, Ventricular fibrillation, Atrial fibrillation

## Abstract

**Background:**

Continuous-flow left ventricular assist devices (LVADs) improve survival and morbidity in patients with stage D heart failure. Management of LVADs for longer durations is necessary in some clinical settings, and a better understanding of the hemodynamics of patients using LVADs is warranted. Arrhythmia, including atrial (AA) and ventricular (VAs) arrhythmias, is a modifying factor of hemodynamics that is highly prevalent among patients with LVADs. However, the clinical impact of arrhythmias in various clinical settings in patients with LVAD, in which the hemodynamic load is likely to present as worsening of right heart failure, remains to be completely elucidated.

**Case presentation:**

We describe the case of a patient under sustained ventricular fibrillation for extraordinarily long duration who was stabilized using LVAD support and in whom newly developed atrial fibrillation led to a significant worsening of right heart failure while using an LVAD.

**Conclusion:**

This case demonstrates the substantial clinical impact of AAs in the management of right heart failure using an LVAD.

**Electronic supplementary material:**

The online version of this article (10.1186/s12872-019-1132-1) contains supplementary material, which is available to authorized users.

## Background

In patients with medically intractable heart failure, continuous-flow left ventricular assist devices (LVADs) improve quality of life as well as survival and morbidity rates compared with conventional medical therapy [1]. Although LVAD use improves the hemodynamic derangement, such as decreased output, triggered by impaired left ventricular systolic function or mitral regurgitation, atrial arrhythmias (AAs) and ventricular arrhythmias (VAs), both highly prevalent in LVAD patients, are considered as poor prognostic factors [2]. During ventricular fibrillation (VF), it is sometimes possible to maintain hemodynamic stability using LVADs, however, the hemodynamic characteristics of these arrhythmias under LVADs have not been fully elucidated. In particular, the association between right heart failure and the development of arrhythmias in patients with LVADs remains unclear. We report the case of a patient with sustained VF for 3 years under LVAD support who had worsening of heart failure with new onset of atrial fibrillation (AF).

### Case presentation

A 47-year-old male developed heart failure due to dilated cardiomyopathy 12 years ago. A cardiac resynchronization therapy-defibrillator (CRT-D; Medtronic^®^ Viva XT CRT-D; AAI 60) was implanted due to VF 7 years ago, and as a bridge to transplantation, a HeartMate II® LVAD was implanted 4 years ago. No arrhythmia developed immediately after LVAD implantation; thus, his CRT-D shock therapy was turned off immediately after LVAD implantation. At the time of LVAD implantation, his transthoracic echocardiographic study showed a significant decrease in the left ventricular (LV) contractility (ejection fraction; 13%), dilation of left ventricle (51 mm in diastole) and trivial aortic regurgitation (AR) without opening of aortic valve but right ventricular (RV) contraction had maintained well relatively (RV fractional area change; 33%).

Eight months after LVAD implantation, the patient developed palpitations and was admitted to our hospital due to repeated VAs necessitating electrical defibrillation. Echocardiography showed the left ventricle diameter did not change, whereas right ventricle volume was slightly enlarged. The repeated VAs were also refractory to various anti-arrhythmic agents, including amiodarone, nifekalant, mexiletine and lidocain, with eventual progression to sustained VF. The hemodynamic compromise due to sustained VF resulted in liver congestion, which was alleviated with a phosphodiesterase type 5 inhibitor, diuretics, and rotation speed optimization (from 8800 to 9600 rpm). These interventions reduced organ dysfunction, suggesting that minimum-required perfusion to vital organs was maintained even under sustained VF. The patient was followed up on an outpatient basis thereafter.

**Fig. 1:**
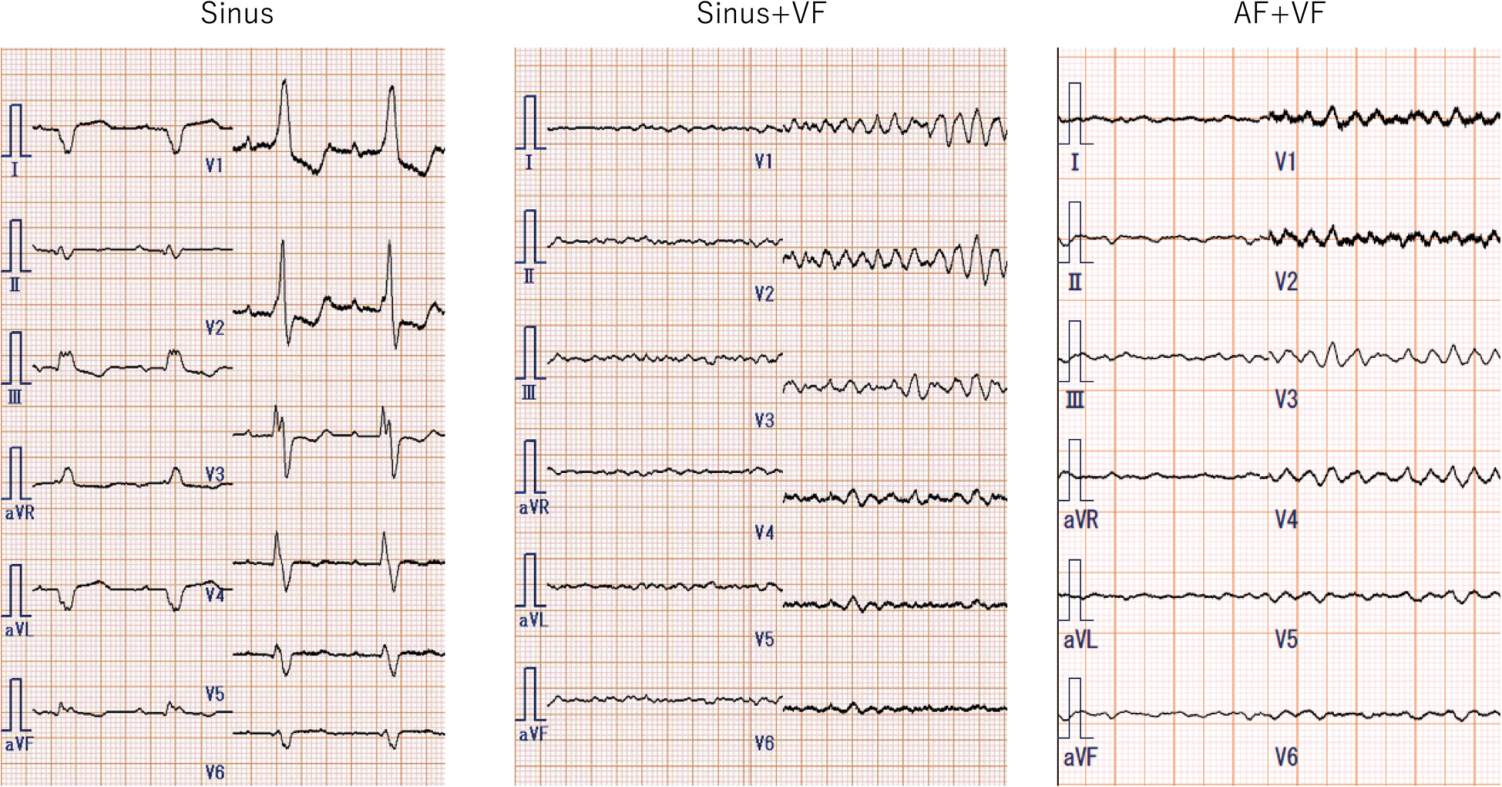
The electrocardiogram of sinus rhythm, ventricular fibrillation (atrial rhythm; sinus rhythm) and ventricular fibrillation (atrial rhythm; atrial fibrillation). VF: ventricular fibrillation

**Fig. 2:**
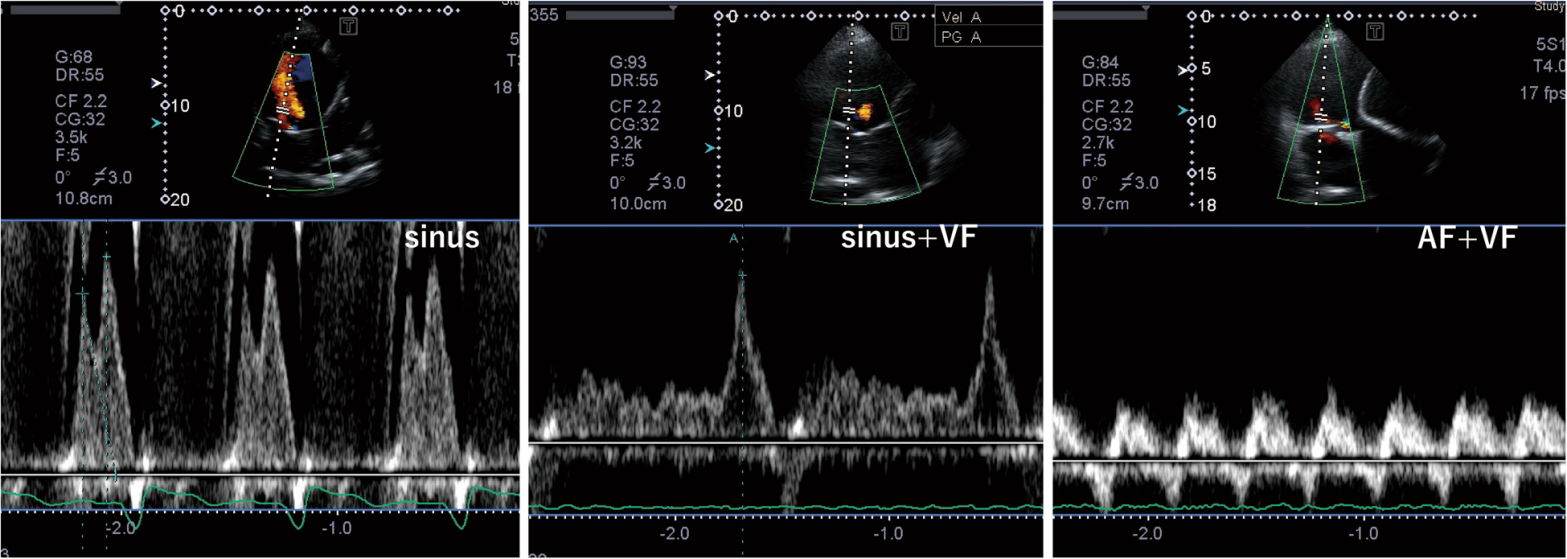
Pulse-wave Doppler showing transmitral flow wave in each rhythm. Normal mitral valve E and A waves were observed in sinus rhythm, whereas only mitral valve A wave could be observed during sinus rhythm and ventricular fibrillation (sinus + VF). During atrial and ventricular fibrillation (AF + VF), no obvious E or A waves were observed; however, small fibrillating waves were detected. Abbreviations: AF: atrial fibrillation, VF: ventricular fibrillation

**Fig. 3:**
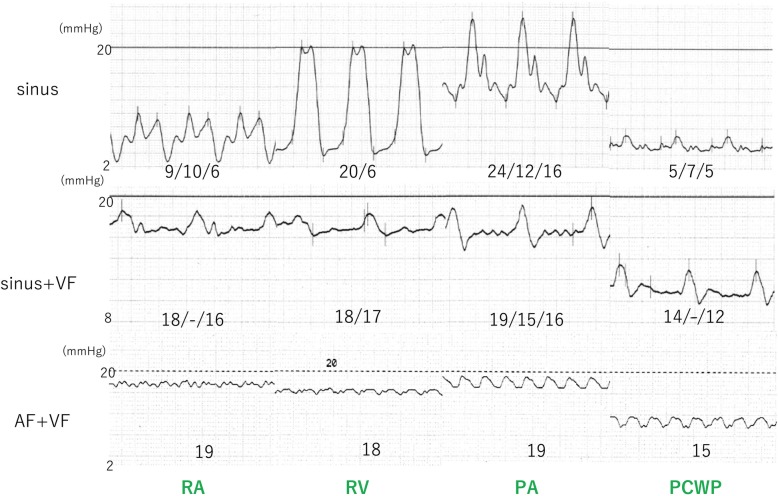
Hemodynamic study with catheterization of the right heart during different rhythms, including sinus rhythm (sinus), sinus rhythm and ventricular fibrillation (sinus + VF), and atrial and ventricular fibrillation (AF + VF). Pressure waves of the right atrium (RA) were presented as “a wave / v wave / mean pressure”. Pressure waves of right ventricle (RV) were presented as “systolic / end-diastolic pressure”. Pressure waves of pulmonary artery (PA) were presented as “systolic / diastolic / mean pressure”. Pressure waves of pulmonary artery wedge pressure (PAW) were presented as “a wave / v wave / mean pressure”. The timing of systole or diastole in VF could not be determined, so that highest and lowest pressures were presented in PA and RV during VF

**Fig. 4:**
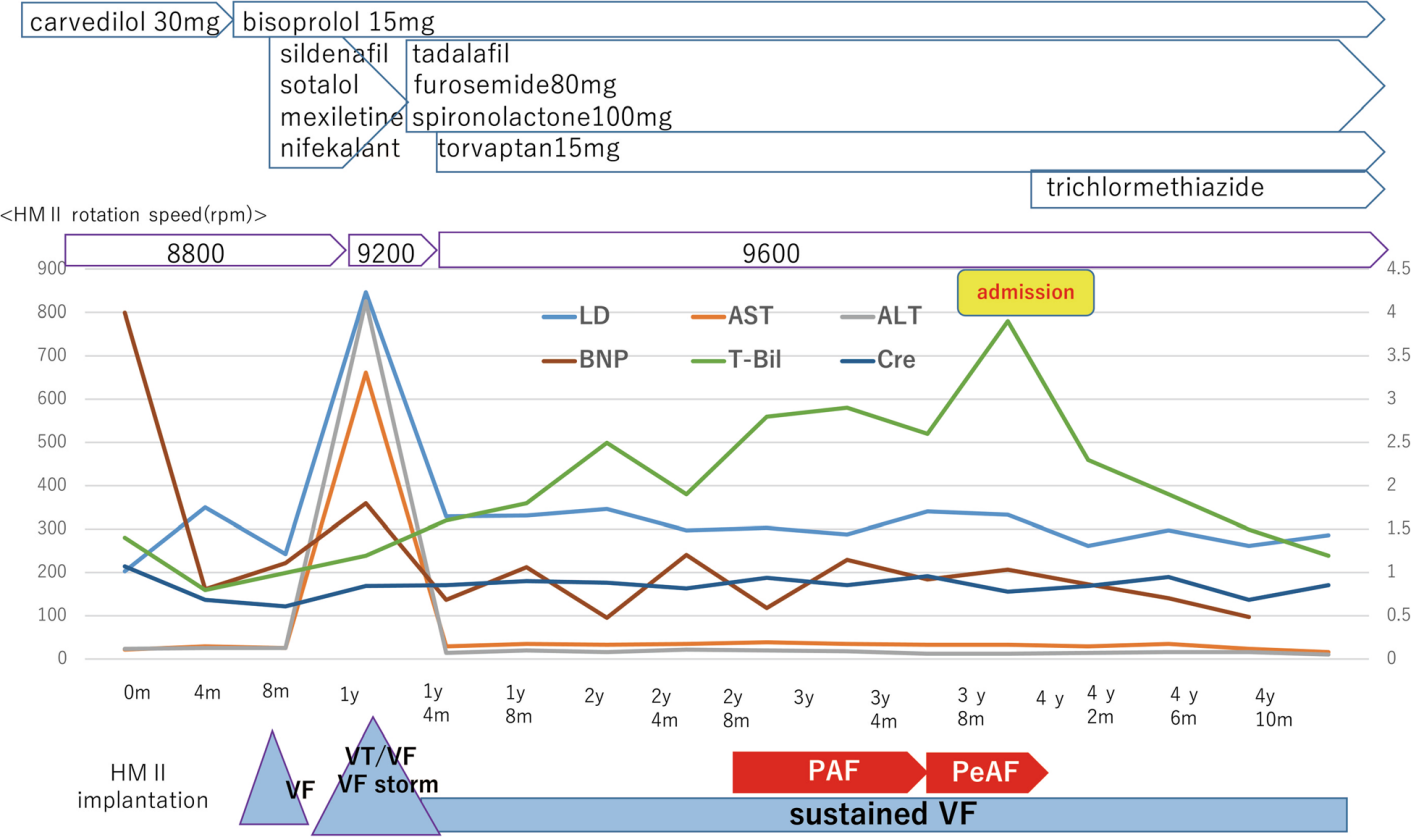
Time course of laboratory data including lactate dehydrogenase (LD), aspartate aminotransferase (AST), alanine aminotransferase (ALT), B-natriuretic peptide (BNP), total bilirubin (T-Bil), and creatinine (Cre) after LVAD implantation. Medications were recorded and each arrhythmic event was described. Abbreviations: VT; ventricular tachycardia, VF; ventricular fibrillation, PAF; paroxysmal atrial fibrillation, PeAF; persistent atrial fibrillation, HMII; HeartMate II


Approximately 2 years after the development of sustained VF, paroxysmal AF was detected on the monitoring records of CRT-D, with a gradually increasing frequency. After 3 years of sustained VF, the patient was readmitted to our hospital due to worsening of symptoms associated with right heart failure and liver congestion (total bilirubin, 3.9 mg/dl). Although his electrocardiogram remained sustained VF (Fig. 1), the CRT-D revealed conversion of the sinus or atrial pacing rhythm to persistent AF. Transthoracic echocardiography revealed that fibrillation of the atrium resulted in the disappearance of not only the mitral flow but also the RV outflow tract doppler flow by the atrial kick (Fig. 2, Additional file 1, 2, 3, 4, 5 and 6). Under sustained VF, RV cardiac output is greatly dependent on atrial kick in which the contribution of atrial kick extraordinary enhanced. The hemodynamic study indicated that the pressure wave from the right atrium (RA) to the right ventricle was significantly flattened resulting that the pulmonary artery pulsatility index, which is defined as the ratio of pulmonary artery pulse pressure to right atrial pressure, was markedly decreased. It suggested a marked reduction in blood flow induced by RA contraction in persistent AF as opposed to sinus rhythm (Fig. 3). As the incremental rotation speed lead to an increase in RA pressure (RAP) from 19 mmHg to 22 mmHg, the rotation speed was set to 9600 rpm and the right heart congestion was treated with additional diuretics insufficiently and the enhanced level of total bilirubin was prolonged. Until four months later, heart failure was gradually improved and the level of total bilirubin decreased below 2 mg/dl. The monitoring records of CRT-D revealed the recovery of sinus rhythm during previous four months (Fig. 4). Although he had remained sustained VF, the recovery of sinus rhythm finely corresponded to the improvement of heart failure and the level of his total bilirubin decreased to 1.2 mg/dl. It intensely corroborated the contribution of persistent AF on the worsening of right heart failure.

**Additional file 1** (MOV 108 kb)



**Additional file 2** (MOV 87 kb)



**Additional file 3** (MOV 148 kb)



**Additional file 4** (MOV 147 kb)



**Additional file 5** (MOV 133 kb)



**Additional file 6** (MOV 128 kb)


## Discussion and conclusions

Both AAs and VAs are common in patients with severe heart failure under LVAD support; furthermore, VAs have been reported in about 20% of LVADs patients [3], with some of them being refractory to various therapies. AAs are also common in patients under LVAD support and are associated with increased readmission and mortality rates [4]. In Japan, long-term LVAD use has become a necessity due to an extreme shortage of donor hearts, requiring a more efficient management of such arrhythmia-related issues [5].

We have reported the case of a patient who developed VAs 8 months after LVAD implantation and sustained VF for the following 4 years. To date, there have been several case reports revealing the tolerability of sustained VF under LVADs; furthermore, we have also experienced some cases of sustained VF without progressive organ dysfunction [5]. However, hemodynamics is not preserved in some patients under sustained VF, and the clinical factors that determine the tolerability of sustained VF remain unknown [6]. The patient in the present case tolerated sustained VF for the longest duration about 3 years reported in the published literature. However, after the onset of AF, the patient gradually developed right heart failure and it was worsened further after he developed persistent AF. The current case suggested the hemodynamic status of VF under LVAD might be determined by other surrounding factors such as atrial function and the change of sinus or atrial pacing rhythm to AF led to the disappearance of regular RA contractions. Consequently, the atrial wave of RAP disappeared and the pressure wave pattern of RAP and RV completely flattened. The subsequent reductions in the RA and RV outputs supposedly led to a worsening of the patient’s right heart failure.

Right atrial contraction contributes to approximately 15–30% of the RV stroke volume; however, its contribution differs across patients with heart failure. The development of AF generally decreases the cardiac output [7], whereas the derangement of AF in cardiac output is somewhat counteracted under LVAD support. Enriquez et al. have demonstrated no significant differences in peak VO_2_ during a cardiopulmonary exercise test between patients with and without AF under LVAD support [2]. Conversely, the incidence of heart failure hospitalization has reportedly increased due to AF, suggesting that the right side of the heart remains sensitive to the hemodynamic effects of AF. A small case series demonstrated an improvement in right heart failure after catheter ablation of AF in patients with LVADs [8]; therefore, rhythm control may be an important strategy for managing right heart failure for this group of patients. If it was difficult to control of our patient’s right heart failure, we considered the catheter ablation of AF. In the present case, the pulmonary artery pulsatility index, which was reported to correspond to RV stroke volume [9], was somewhat maintained in sinus rhythm even under the sustained VF, whereas it significantly decreased by the development of AF. Several clinical conditions of right heart failure enhance the impact of right atrial function [10]. D’Alto et al. have also demonstrated the enhanced impact of right atrial function in idiopathic pulmonary artery hypertension [11]. Specifically, the contribution of AAs is significantly enhanced in sustained VF, in which the fibrillating ventricle has minimal contribution to the systemic output, and the coexistence of AAs and VAs may be critical. The current case highlights the significance of right atrial function as a determinant of hemodynamics in patients with sustained VF under LVAD support and suggests a non-invasive approach for the treatment of heart failure.

According to the risk of thromboembolism, it is complicated in patients with AF under LVAD support. There was a report of thromboembolism risk in patients with AF under LVAD [12], whereas a recent meta-analysis exhibited the presence of AF did not increase the risk of thromboembolism [13].

This case also emphasizes the difficulty of diagnosing AF under sustained VF. The findings on electrocardiogram of patients under sustained VF remain unchanged when AF is newly developed. Therefore, the detection of AF may be difficult, and monitoring of device records, mitral flow visualization using echocardiography or visualization of pressure wave by Swan-Ganz catheter is required. The occurrence of newly developed AF under sustained VF is a rare case; however, the understanding of the hemodynamic effect of these arrhythmias under LVAD support is much informative for future cases of LVAD support.

Indeed, AF might be a sign or symptom of a deterioration of HF, however, the risk of new development of AF is complicated under LVAD. For instance, Deshmukh A et al. demonstrated paroxysmal AF was improved after LVAD implantation due to improvement of electroanatomical remodeling [14]. On the other hand, LVAD related complications such as AR and right heart failure might also impose the excessive burden to atrium leading to AF progression. The determining factor of AF progression in the current study was beyond the scope of the current case report.

We report the case of a patient with sustained VF for 3 years under LVAD support who had worsening of heart failure with new onset of AF. The hemodynamic characteristics of stabilized sustained VF under LVADs have not been fully elucidated, however this case showed the contribution of AAs is significantly enhanced in sustained VF.

## Additional files

Changes in the left ventricular (LV) and left atrial (LA) contraction as well as in blood flow in the tricuspid valve during each rhythm [1–2: sinus rhythm, 3–4: sinus rhythm and ventricular fibrillation (sinus + VF), 5–6: atrial fibrillation and ventricular fibrillation (AF + VF)]. In the sinus rhythm, LV and LA contracted regularly in turn (1) and rhythmic blood flow in the tricuspid valve was observed (2). There was no significant tricuspid valve regurgitation. In the sinus + VF rhythm, LA contracted regularly, whereas no visible contractions were observed by fibrillating LV (3, 4). The regular mitral and tricuspid valve movements were also observed. In the AF + VF rhythm, no regular contractions of LV and LA were observed (5) and blood flow to and from the tricuspid valve was detected (6). The regular mitral and tricuspid valve movements could not be observed.

## Data Availability

Not applicable.

## Abbreviations

AA: Atrial arrhythmia
AF: Atrial fibrillation
AR: Aortic regurgitation
LV: Left ventricle
LVAD: Left ventricular assist devices
RA: Right atrium
RAP: Right atrial pressure
RV: Right ventricle
VF: Ventricular fibrillation
